# Targeting lysyl oxidase for molecular imaging in breast cancer

**DOI:** 10.1186/s13058-015-0609-9

**Published:** 2015-08-13

**Authors:** Melinda Wuest, Manuela Kuchar, Sai Kiran Sharma, Susan Richter, Ingrit Hamann, Monica Wang, Larissa Vos, John R. Mackey, Frank Wuest, Reik Löser

**Affiliations:** Department of Oncology, University of Alberta, 11560 University Avenue, Edmonton, AB T6G 1Z2 Canada; Helmholtz-Zentrum Dresden-Rossendorf, Institute of Radiopharmaceutical Cancer Research, Bautzner Landstrasse 400, 01328 Dresden, Germany; Faculty of Pharmacy and Pharmaceutical Sciences, University of Alberta, 11361 87 Avenue, Edmonton, AB T6G 2E1 Canada

## Abstract

**Introduction:**

Lysyl oxidase (LOX; ExPASy ENZYME entry: EC 1.4.3.13) and members of the LOX-like family, LOXL1–LOXL4, are copper-dependent enzymes that can modify proteins of the extracellular matrix. Expression of LOX is elevated in many human cancers, including breast cancer. LOX expression correlates with the level of tissue hypoxia, and it is known to play a critical role in breast cancer metastasis. The goal of the present study was to target LOX with (1) molecular probe fluorescent labeling to visualize LOX in vitro and (2) a radiolabeled peptide to target LOX in vivo in three different preclinical models of breast cancer.

**Methods:**

Gene expression of all five members of the LOX family was analyzed at the transcript level via microarray analysis using tissue biopsy samples from 176 patients with breast cancer. An oligopeptide sequence (GGGDPKGGGGG) was selected as a substrate-based, LOX-targeting structure. The peptide was labeled with fluorescein isothiocyanate (FITC) for confocal microscopy experiments with the murine breast cancer cell line EMT-6. In vivo molecular imaging experiments were performed using a C-terminal amidated peptide, GGGDPKGGGGG, labeled with a short-lived positron emitter, fluorine-18 (^18^F), for positron emission tomography (PET) in three different breast cancer models: EMT6, MCF-7 and MDA-MB-231. The PET experiments were carried out in the presence or absence of β-aminopropionitrile (BAPN), an irreversible inhibitor of LOX.

**Results:**

Immunostaining experiments using a LOX-specific antibody on EMT-6 cells cultured under hypoxic conditions confirmed the elevation of LOX expression in these cells. An FITC-labeled oligopeptide, FITC-Ava-GGGDPKGGGGG-NH_2_, was found to be localized in different cellular compartments under these conditions. After injection of [^18^F]fluorobenzoate-GGGDPKGGGGG-NH_2_, radioactivity uptake was visible in all three breast cancer models in vivo. Tumor uptake was reduced by predosing the animals with 2 mg of BAPN 4 h or 24 h before injection of the radiotracer.

**Conclusions:**

The present data support further investigation into the development of LOX-binding radiolabeled peptides as molecular probes for molecular imaging of LOX expression in cancer.

## Introduction

Breast cancer is the most common malignancy in women worldwide [1]. The high degree of diversity in the molecular profile makes clinical management and treatment of breast cancer a special challenge [2]. Despite continued progress and innovation in early detection of breast cancer, 30 % of patients treated for localized breast cancer develop recurrence of the disease at distant sites [3]. Tumor progression and metastatic spread are complex, multistep processes involving dynamic interactions between tumor cells, stromal cells and the extracellular matrix (ECM). Hypoxia and ECM are two major non-cellular components of the tumor microenvironment that influence metastasis [4].

The family of lysyl oxidase (LOX) enzymes is a central player in remodeling of cancer-related ECM. All members of the LOX family are copper-dependent amine oxidases that consist of five paralogs: LOX and LOX-like 1–4 (LOXL1–LOX4) [5]. Their primary function is the covalent cross-linking of different types of collagen and elastin, two basic components of the ECM that ensure structural integrity of many tissues [6].

LOX is synthesized as a pre-proenzyme that is cleaved from the endoplasmic reticulum before glycosylation of the formed N-terminal propeptide and subsequent folding of the C-terminal end. After incorporation of copper into the catalytic site, the proenzyme is released into the extracellular space [5].

Initially proposed to act mainly as a tumor suppressor, members of the LOX enzyme family are gaining relevance in their role as promoters of tumor progression and metastasis [7–9]. This makes the LOX family an ideal target for treatment of metastatic disease. Altered levels of LOX expression were found in a number of human malignancies, including breast, colorectal, pancreatic, lung and prostate cancers [9].

Most studies have been conducted in various breast cancer models to assess potential roles of the LOX enzyme family as molecular targets in the development of novel therapeutic drugs [10]. Hypoxic breast cancer cells produce elevated levels of LOX, which may play a critical role in tumor progression and metastasis [11–13]. Inhibitors of LOX function are small molecules, small interfering RNAs, oligopeptides and antibodies [14–20]. The most prominent inhibitor of LOX function is the small-molecule inhibitor β-aminopropionitrile (BAPN) [15, 21]. Other small-molecule inhibitors of LOX include bioreductively activated BAPN derivatives [20]. BAPN irreversibly blocks LOX enzyme activity, leading to the resynthesis and release of LOX. Use of BAPN in various preclinical breast cancer models led to a significant reduction of primary tumor volume and number of metastases [22, 23], but it also caused systemic side effects [24].

Functional molecular imaging of LOX enzyme expression in vivo would open novel opportunities for non-invasive detection, staging and monitoring therapy of cancer. Positron emission tomography (PET) has been used for numerous diagnostic and prognostic applications in breast cancer imaging, including the monitoring of treatment response [25–30]. In PET, short-lived radiotracers (e.g., fluorine-18 [^18^F]) are used to allow for real-time dynamic monitoring of biochemical processes, such as metabolic and proliferative activity at the molecular level.

PET imaging of in vivo expression of LOX enzymes requires the development of LOX enzyme-targeting radiotracers. Nagan and Kagan have studied various lysine-containing oligopeptides derived from N-terminal type I collagen telopeptide for their LOX-catalyzed conversion [31]. They identified the oligopeptide Ac-GGGDPKGGGGG-NH_2_ as a substrate with favorable kinetic properties for LOX-mediated peptidyl lysine oxidation. This makes the peptide sequence GGGDPKGGGGG a suitable lead targeting vector for the development of a PET radiotracer for molecular imaging of LOX. Introduction of ^18^F into peptides is a well-established radiolabeling technique, and we recently reported the radiolabeling of GGGDPKGGGGG via acylation with the prosthetic group *N*-succinimidyl-4-[^18^F]fluorobenzoate ([^18^F]SFB) [32].

The goal of the present work was threefold: (1) verification of the LOX enzyme family as a promising molecular target in breast cancer by analysis of LOX enzyme expression in human breast cancer and normal breast tissue samples, (2) in vitro study of LOX expression and analysis of the fluorescently labeled LOX-targeting peptide fluorescein isothiocyanate (FITC)-Ava-GGGDPKGGGGG-NH_2_ in the EMT-6 murine breast cancer cell line and (3) analysis of the in vivo radiopharmacological profile of the ^18^F-labeled oligopeptide [^18^F]fluorobenzoate ([^18^F]FB)-GGGDPKGGGGG-NH_2_ in murine (EMT-6) and human (MCF-7 and MDA-MB-231) breast cancer models using PET.

## Methods

### Patient samples

Gene expression microarray analysis was performed on primary samples from 176 treatment-naive patients with breast cancer and on 10 healthy breast tissue samples collected from reduction mammoplasties through the Canadian Breast Cancer Foundation Tumor Bank (Northern Alberta Study Center, Cross Cancer Institute, Edmonton, AB, Canada). Institutional ethical approval was obtained from the Alberta Cancer Research Ethics Committee. Patient information was collected under Research Ethics Board protocol ETH-02-86-17. All necessary consents from all patients involved in this study were obtained according to the aforementioned ethics protocol. The tumor samples, collected at surgery, were frozen in liquid nitrogen within 20 min after collection. Histological analysis of the frozen samples allowed the differentiation of neoplastic and benign tissue and indicated that at least 70 % of the cells present were invasive tumor cells. Total RNA was isolated from the frozen samples using TRIzol reagent (Life Technologies, Carlsbad, CA, USA) and RNeasy columns (Qiagen, Valencia, CA, USA). The RNA was quantified using a NanoDrop 1000 spectrophotometer (NanoDrop/Thermo Scientific, Wilmington, DE, USA), and its integrity was evaluated using a 2100 Bioanalyzer (Agilent Technologies, Santa Clara, CA, USA). RNA samples with RNA integrity numbers greater than 7.0 were used. The RNA was subjected to linear amplification and cyanine 3 labeling, then hybridized to Agilent Technologies whole human genome arrays using Agilent Technologies kits (One Color Low RNA Input Linear Amplification Kit Plus, One Color RNA Spike-In Kit and Gene Expression Hybridization Kit). The arrays were scanned using an Agilent Technologies scanner. The data were extracted and quality was evaluated using Feature Extraction 9.5 software, and they were normalized and analyzed using GeneSpring GX 7.3 software (both from Agilent Technologies). Data for LOXL3 and LOXL4 represent combined data because two different oligonucleotides were found.

The data used in this publication have been deposited in the U.S. National Center for Biotechnology Information Gene Expression Omnibus (GEO) and are accessible through series accession numbers [GEO:GSE22820 and [GEO:GSM741698] [33].

### Peptide syntheses

#### General

Peptide synthesis reagents were purchased from Novabiochem (EMD Millipore, Billerica, MA, USA). Fluorescein-5-thiourea-Ava-GGGDPKGGGGG-NH_2_ was synthesized using standard orthogonal fluorenylmethyloxycarbonyl-based solid-phase peptide synthesis (SPPS) on a Syro I peptide synthesizer (MultiSynTech/Biotage, Witten, Germany) or a Liberty peptide synthesizer combined with the Discover SP microwave reactor (CEM, Matthews, NC, USA). “Ava” represents the linker moiety 5-aminovaleric acid; G, D, P and K are the L-amino acids glycine, aspartic acid, proline and lysine. The FITC label was coupled manually using an on-resin approach. Mass spectra were recorded on a 6220 orthogonal acceleration time-of-flight mass spectrometer system (Agilent Technologies) with electron spray ionization (ESI) mass spectrometry (MS). Semipreparative and analytical high-performance liquid chromatography (HPLC) experiments were performed using a Gilson system (Gilson, Middleton, WI, USA) with a Jupiter Proteo 10-μm 90 Å, 250×10-mm, 4.5-μm C18 reversed-phase column (Phenomenex, Torrance, CA, USA). Ultraviolet absorbance was monitored at 210- and 254-nm wavelengths. The mobile phase consisted of water/0.2% trifluoroacetic acid (TFA) as solvent A and acetonitrile as solvent B.

#### Fluorescein 5-thiourea Ava-GGGDPKGGGGG NH_2_

**Fig. 1:**
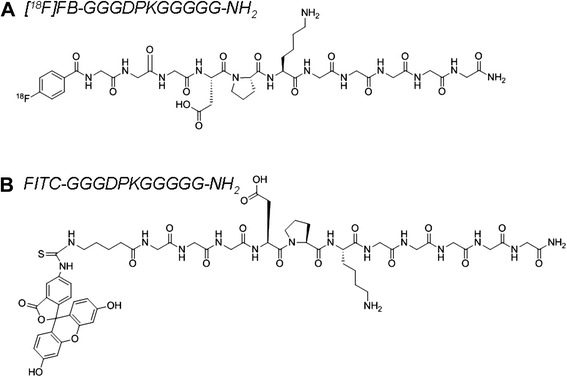
Chemical structures of (**a**) the fluorine-18-labeled peptide [^18^F]fluorobenzoate ([^18^F]FB)-GGGDPKGGGGG-NH_2_ and (**b**) the fluorescein isothiocyanate (FITC)-labeled peptide FITC-GGGDPKGGGGG-NH_2_

The fluorescein 5-thiourea-Ava-GGGDPKGGGGG NH_2_ peptide amide (Fig. 1) was synthesized starting from 100-mg Rink amide 4-methylbenzhydrylamine (MBHA) resin (loading, 0.6 mmol/g), including the N-terminal Ava residue, via automated SPPS [34]. The fluorescein label was coupled on-resin using FITC and standard protocols. Briefly, the peptidyl resin was incubated with 1.2 Eq of FITC and 1.2 Eq of triethylamine/dimethylformamide (DMF) (3 ml/0.1 mmol resin-bound peptide) solution overnight under exclusion of light. Total cleavage was performed with TFA-H_2_O-triisopropylsilane (TIPS) at a 95:4:1 ratio followed by precipitation of the bright orange crude peptide with ice-cold diethylether (47 % crude yield). The pure peptide was obtained after semipreparative HPLC purification with a flow rate of 2 ml/min and a gradient of 0–10 min 10 % B, 25 min 50 % B, 30–35 min 70 % B, 40 min 90 % B (*t*_R_ = 29.5 min) and lyophilization as an orange powder (30 mg, 25 μmol, 42 % yield). The molecular weight of C_57_H_71_N_15_O_19_S was calculated as 1301.5 g/mol and found through low-resolution mass spectrometry (ESI, positive) as 1302.5 g/mol [M + H] + and 651.7 g/mol [M + 2H]^2+^.

#### Radiosynthesis of [^18^F]FB-GGGDPKGGGGG-NH_2_

Radiosynthesis of [^18^F]FB-GGGDPKGGGGG-NH_2_ (Fig. 1) was carried out according to the method described by Kuchar et al. [32], which is similar to the SPPS of ^18^F-labeled peptides developed earlier by Sutcliffe and coworkers [35]. Briefly, 12 mg of Rink amide MBHA resin-bound GGGDPKGGGGG peptide was reacted with 100 μl of [^18^F]SFB in DMF and in 150 μl of 0.05 M sodium hydrogen phosphate buffer at pH 9. The reaction mixture was heated for 30 min at 50 °C. The resin was washed with simultaneous removal of the protecting group, and resin cleavage was carried out for 20 min at room temperature using TFA-H_2_O-TIPS at a 95:4:1 ratio. The final product, [^18^F]FB-GGGDPKGGGGG-NH_2_, was obtained after a total synthesis time of 120 min, including HPLC purification and isolation of the product. The radiochemical yield was 18–50 % (decay-corrected), and the radiochemical purity exceeded 98 %.

### Cell culture

Murine EMT-6 (gift from Dr. David Murray, Department of Oncology, University of Alberta, Edmonton, AB, Canada), human MCF-7 and human MDA-MB-231 breast cancer cell lines (American Type Culture Collection, Manassas, VA, USA) were grown in a CO_2_ incubator at 37 °C, in Gibco Dulbecco’s modified Eagle’s medium/Nutrient Mixture F-12 (Life Technologies) supplemented with 15 mM *N*-2-hydroxyethylpiperazine-*N*-2-ethanesulfonic acid (Gibco catalog number 15630; Life Technologies), 2 mM l-glutamine, 10 % fetal bovine serum (FBS, Gibco catalog number 12483; Life Technologies) and 1 % penicillin-streptomycin in T75 flasks with media renewed every 2–3 days.

### Confocal microscopy

EMT-6 cells were harvested at 80 % confluence using 0.25 % trypsin- ethylenediaminetetraacetic acid (EDTA) (Gibco catalog number 25200; Life Technologies), plated onto sterilized glass coverslips and placed in autoclaved Petri dishes at a density of 2.5×10^5^ cells per plate. Cells on coverslips were allowed to incubate for 24 h under sterile normoxic versus hypoxic conditions achieved by using a specialized sealed chamber device to replace O_2_ with N_2_ [36].

Cells were rinsed thrice with phosphate-buffered saline (PBS) and incubated with anti-LOX rabbit polyclonal immunoglobulin G (IgG) primary antibody (1:500 dilution, catalog number sc-66947; Santa Cruz Biotechnology, Santa Cruz, CA, USA) in complete growth media for 1 h at 37 °C. Cells were washed thrice with PBS before incubation with goat anti-rabbit IgG-CFL 488 secondary antibody (1:1000 dilution, catalog number sc-362262; Santa Cruz Biotechnology) for 1 h at 37 °C. In a separate set of experiments, FITC-Ava-GGGDPKGGGGG-NH_2_ was incubated with the cells at a concentration of 25 μM for 15 min to observe direct fluorescence. Cells were rinsed thrice with PBS and fixed with 4 % paraformaldehyde (catalog number 158127; Sigma-Aldrich, Oakville, ON, Canada) in PBS (pH 7.4) at room temperature for 15 min.

Cells were finally rinsed three times with PBS before being mounted on microscopic slides (Fisherfinest 12-544-2, 25×75×1 mm; Fisher Scientific, Pittsburgh, PA, USA) using 40-μl drops of polyvinyl alcohol–based mounting media supplemented with 4′,6-diamidino-2-phenylindole (DAPI; 50 μg/ml). Appropriate blanks and controls were included in the experiments. Cells were imaged using corresponding lasers at optimal fixed wavelength parameters for visualizing DAPI (blue nuclear staining) and IgG-CFL 488 (green emission) through a Plan-Apochromat ×40/1.30 Oil DIC M27 lens on a Zeiss LSM 710 confocal laser scanning microscope (Carl Zeiss Microscopy, Oberkochen, Germany). Images were processed using ZEN 2011 software (Carl Zeiss Microscopy).

### Flow cytometry

EMT-6 cells were plated onto sterilized 60-mm glass dishes. After 24 h, the dishes were divided into two groups and incubated for another 24 h under normoxic versus hypoxic conditions using specialized sealed chamber devices to replace O_2_ with N_2_ [36]. Cells were harvested using a cell scraper and counted. Normoxic and hypoxic cells were pooled and distributed, applying 1×10^6^ cells for each sample. Appropriate blanks and controls were included in all experiments. Cells were rinsed twice with ice-cold fluorescence-activated cell sorting (FACS) buffer (0.5 % FBS, 0.05 % NaN_3_ and 2 mM EDTA in PBS) and incubated with anti-LOX rabbit polyclonal IgG primary antibody (1:20 dilution, catalog number sc-66947; Santa Cruz Biotechnology) in FACS buffer for 30 min at room temperature. Cells were washed three times with FACS buffer and incubated with goat anti-rabbit IgG-CFL 488 secondary antibody (1:100 dilution, catalog number sc-362262; Santa Cruz Biotechnology) for 30 min at room temperature in the dark. In samples treated with FITC-Ava-GGGDPKGGGGG-NH_2_, cells were incubated at a concentration of 25 μM for 60 min at room temperature in the dark. Cells were rinsed three times with FACS buffer and kept on ice until analysis using a FACSCalibur flow cytometer (BD Biosciences, San Jose, CA, USA). Data were acquired from at least 10,000 cells per sample and analyzed with the manufacturer’s software (BD Biosciences).

### In vitro cell uptake of [^18^F]FB-GGGDPKGGGGG-NH_2_

EMT-6 cells were cultured at standard conditions as described above. Using glass cell culture dishes with a diameter of 55 mm, cells were plated at a density of approximately 500,000 cells per plate and incubated for 48 h under normal conditions at 37 °C and 5 % CO_2_. Following that step, half of the plates were taken to culture the cells under hypoxic conditions over the next 24 h, and the others were kept under normal cell culture conditions. Hypoxic cell culture conditions were achieved using specialized sealed chamber devices to replace O_2_ with N_2_ (see above). For the radiotracer incubation, the medium was replaced with Krebs-Ringer buffer (115 mM NaCl, 5.9 mM KCl, 1.2 mM MgCl_2_, 1.2 mM NaH_2_PO_4_, 1.2 mM Na_2_SO_4_, 2.5 mM CaCl_2_, 25 mM NaHCO_3_ and 5 mM glucose at pH 7.4) at room temperature. Approximately 1 MBq of [^18^F]FB-GGGDPKGGGGG-NH_2_-containing buffer was added to each plate and incubated for 10, 30 and 60 min under either normoxic or hypoxic conditions. After incubation, cells were rinsed three times with ice-cold Krebs-Ringer buffer to stop further cell uptake and then immediately lysed using 1 ml of 5 % trichloroacetic acid (TCA) for 1 h. One plate representing each condition was used for protein quantification using the bicinchoninic acid (BCA) protein assay kit (Pierce Biotechnology/Thermo Scientific, Rockford, IL, USA) according to the manufacturer’s recommendations, and bovine serum albumin was used as the protein standard. Following the 1-h lysis with TCA, the supernatants were collected and placed into scintillation vials to be counted using a gamma counter (Wallac Wizard 1480 3; PerkinElmer, Woodbridge, ON, Canada) to determine radioactivity uptake levels in the cells. Radiotracer cell uptake levels were normalized to the percentage of the total added radioactivity per milligram of protein. The data are presented as mean ± standard error of the mean (SEM).

### Animal tumor models

All animal experiments were carried out in accordance with guidelines of the Canadian Council on Animal Care and were approved by the local animal care committee of the Cross Cancer Institute.

Murine EMT-6 cells (5 × 10^6^ cells in 100 μl of PBS) were injected into the upper left flank of female BALB/c mice (20–24 g; Charles River, Saint-Constant, QC, Canada). The EMT-6 tumor-bearing mice were imaged and used for ex vivo biodistribution experiments after allowing 7–10 days for the tumors to reach sizes of about 600 mm^3^.

Human MCF-7 cells, which form xenografts in female athymic mice, were injected subcutaneously (2–5×10^6^ cells in 100 μl of PBS) into 8–10-week-old female NIH-III nu/nu mice (Charles River, Wilmington, MA, USA). Before injection of the cells, all mice received a 0.72-mg pellet containing 17β-estradiol in a 60-day release preparation (Innovative Research of America, Sarasota, FL, USA).

The pellet was implanted subcutaneously into the upper right flank to provide a constant level of 17β-estradiol needed by the estrogen receptor (ER)-positive MCF-7 cells. Tumors were imaged 20–30 days after injection, with the tumors reaching sizes ranging from 200 to 400 mm^3^. Human MDA-MB-231 cells (1×10^6^ cells in 100 μl of PBS) were also injected subcutaneously into 8–10-week-old female NIH-III nu/nu mice. After 7–9 days, they reached a size of approximately 500 mm^3^ and were suitable for the PET experiments.

### Protein expression analysis

Western blots for detection of LOX expression were carried out in cells and tumor tissue of EMT-6, MDA-MB-231 and MCF-7 cells. The cells were grown in 6-well plates to approximately 80 % confluence, washed with PBS, harvested using 100 μl/well of CelLytic™ solution with protease inhibitor cocktail added (both from Sigma-Aldrich) and collected in tubes. The tumor tissue was washed in PBS, minced and lysed in tubes using 100 μl of CelLytic™ solution and protease inhibitor per 25 mg of tumor tissue. After cell lysis on ice for 30 min, whole extracts were centrifuged at 13,500 × *g* for 5 min at 4 °C to remove cell and tumor debris. Protein determination was conducted using a BCA-based protein assay (Pierce Biotechnology/Thermo Scientific). Aliquots of the supernatants were mixed with a 1/4 volume of 4× Laemmli buffer (250 mM Tris-HCl, 8 % (w/v) SDS, 40 % glycerol, 200 mM dithiothreitol and 0.04 % (w/v) bromophenol blue, pH 6.8) and heated for 10 min at 99 °C. The protein extracts were applied to SDS-PAGE gels and separated by electrophoresis. Proteins were transferred to nitrocellulose membranes by electroblotting and blocked for 1 h at room temperature in 5 % (w/v) non-fat dry milk in Tris-buffered saline containing 0.05 % (v/v) Tween 20 (TBST). Membranes were incubated with the following primary antibodies: LOX H140 (1:1000, catalog number sc-66947; Santa Cruz Biotechnology) and rabbit anti-actin (1:1000, catalog number A5420; Sigma-Aldrich) followed by incubation with peroxidase-conjugated anti-rabbit IgG secondary antibodies (1:10,000; Sigma-Aldrich). Incubation with the primary and secondary antibodies was performed in TBST.

### In vivo stability of the radiolabeled peptide

Normal BALB/c mice received injections of approximately 25 MBq of [^18^F]FB-GGGDPKGGGG. Venous blood samples were taken at 5, 15, 30 and 60 min postinjection and processed for determination of metabolic stability in vivo. Blood cells were separated by centrifugation (13,000 rpm for 5 min). Proteins in the supernatant were precipitated by addition of 2 volume parts of methanol followed by a second centrifugation step (13,000 rpm for 5 min). Radioactivity in blood components (cells, proteins and plasma) was measured using a Wallac Wizard 1480 3 automatic gamma counter. The clear plasma supernatant was examined using a Shimadzu HPLC system (Shimadzu Scientific Instruments, Columbia, MD, USA) equipped with a DGU-20A5 degasser, a SIL-20AHT autosampler, a LC-20AT pump, a SPD-M20A photo diode array detector and a RAMONA* radiodetector (Raytest Isotopenmessgeraete, Straubenhardt, Germany). The samples were analyzed using a Phenomenex Luna 10-μm C18(2) 100 Å, 250×4.6-mm column and the following gradients with water/0.2 % TFA as solvent A and acetonitrile as solvent B: 0–3 min 10 % B, 10 min 30 % B, 17 min 50 % B, 23 min 70 % B and 27–30 min 90 % B. The flow was constant at 1 ml/min, and the ultraviolet signals were measured at 210 nm and 254 nm.

### Small animal PET imaging

PET experiments were performed with [^18^F]FB-GGGDPKGGGGG-NH_2_ injected into EMT-6 tumor-bearing BALB/c mice or into NIH-III mice bearing subcutaneous MCF-7 or MDA-MB-231 tumors, and tracer uptake was analyzed in vivo. For radiotracer injection, a catheter was placed into the tail vein of the mouse. While under anesthesia, mice were placed in the prone position with the medial axis parallel to the axial axis of the scanner and the thorax, abdomen and hind legs placed into the center of the field of view of the microPET R4 or Inveon PET/computed tomography (CT) scanner (Siemens Preclinical Solutions, Knoxville, TN, USA). A transmission scan for attenuation correction was not acquired. [^18^F]FB-GGGDPKGGGGG-NH_2_ (4–5 MBq) in 100–150 μl of saline was injected intravenously through the catheter into the tail vein. The effects of BAPN were analyzed after intraperitoneal injection of 2 mg (100 mg/kg) of the inhibitor 4 h and 24 h before radiotracer injection. For the different experimental protocols, either an emission scan 60 min postinjection (dynamic experiments) was performed immediately or a 20 min postinjection PET scan acquisition (static scan followed by CT scan) was performed. Depending on the experimental setup, data acquisition continued for either 60 min or 20 min in three-dimensional list mode. The dynamic list mode data were sorted into sinograms with 53 time frames (10×2 s, 8×5 s, 6×10 s, 6×20 s, 8×60 s, 10×120 s and 5×300 s). The frames were reconstructed using ordered subset expectation maximization or maximum a posteriori reconstruction modes.

The pixel size was 0.085×0.085×0.12 cm, and the resolution in the center field of view was 1.8 mm. Correction for partial volume effects was not performed. The image files were further processed using the ROVER v2.0.51 software (ABX, Radeberg, Germany). Masks defining three-dimensional regions of interest (ROIs) were set, and the ROIs were defined by 50 % thresholding. Mean standardized uptake values (SUV_mean_ = [activity/ml tissue]/[injected activity/body weight], mL/kg] were calculated for each ROI. Time–activity curves (TACs) were generated for the dynamic scans only. All semiquantified PET data are presented as mean ± SEM.

## Results

### LOX and LOXL1–LOXL4 mRNA expression in breast cancer samples

**Fig. 2:**
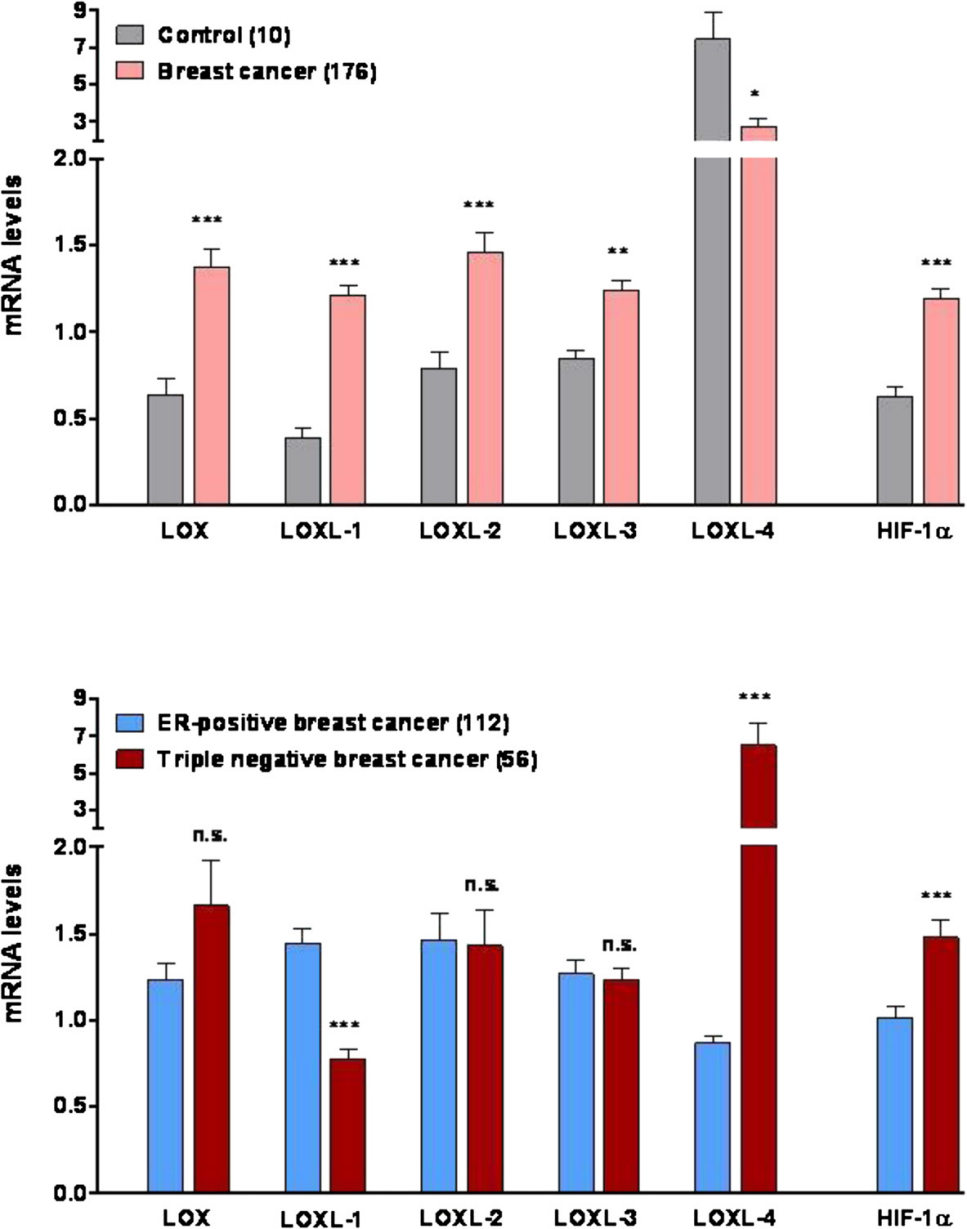
Total mRNA expression of lysyl oxidase (LOX), four members of the LOX-like family (LOXL1– LOXL4) and hypoxia-inducible factor (HIF)-1α in control breast versus breast cancer tissue (*top*) and estrogen receptor (ER)-positive versus triple-negative breast cancer samples (*bottom*). Data are shown as mean ± standard error of the mean of mRNA levels based on log-transformed values of the gene expression microarray signal intensity from analyzed patient samples. *n.s.* not significant, *p<0.05, **p<0.01, ***p<0.001

Figure 2 presents retrospective mRNA expression based on tissue microarray (TMA) analysis of LOX and LOXL1–LOXL4 in breast cancer tissue biopsy samples from 176 patients with breast cancer versus 10 healthy control human breast tissue samples.

The mRNA expression levels of hypoxia-inducible factor hypoxia-inducible factor (HIF)-1α were also analyzed. Except for LOXL4, all enzymes of the LOX family, as well as HIF-1α, were upregulated in breast cancer samples. LOXL4 was found to have higher average mRNA transcript levels in healthy tissue compared with breast cancer samples. Analysis of 56 triple-negative breast cancer (TNBC) versus 112 ER-positive breast cancer samples revealed higher LOX mRNA transcript levels in TNBC than in ER-positive breast cancer. Both LOXL4 and HIF-1α mRNA levels were significantly higher in this breast cancer patient group than in ER-positive samples.

In contrast to that finding, no differences in LOXL2 and LOXL3 mRNA expression levels were found in ER-positive breast cancer and TNBC. Interestingly, LOXL1 showed decreased mRNA levels in TNBC samples. In summary, TNBC samples displayed higher HIF-1α expression levels than other breast cancer samples. In direct comparison with ER-positive breast cancer, TNBC had higher mRNA levels for HIF-1α, LOX and LOXL4. However, LOXL1 mRNA levels in TNBC samples were significantly lower compared with that of ER-positive breast cancer samples. Recent reports in the literature and the present TMA analytical data confirm that LOX is a suitable molecular target for breast cancer therapy. Moreover, LOX could also be an interesting target for the development of PET radiotracers for the molecular imaging of its in vivo expression in breast cancer.

### LOX protein expression in breast cancer cell lines and tumor models in mice

**Fig. 3:**
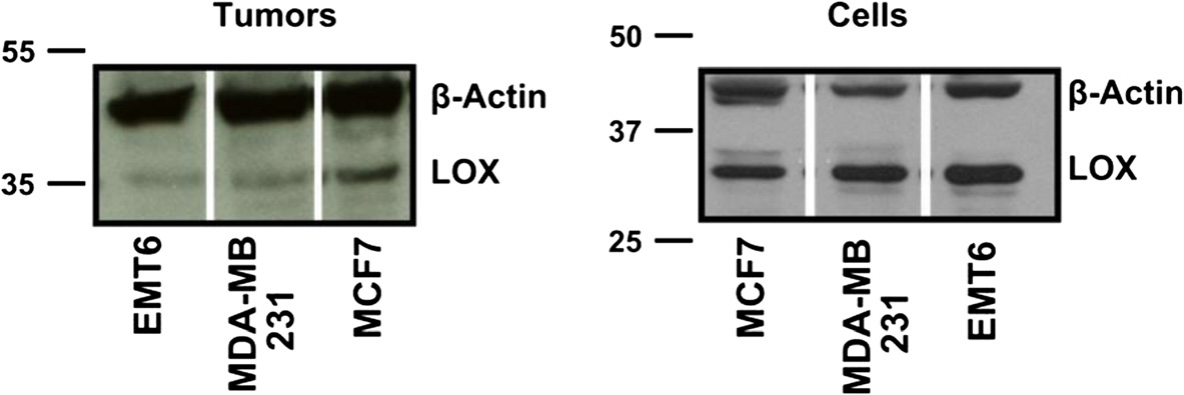
Western blots showing expression of lysyl oxidase (LOX) in its active form (approximately 35 kDa) in all three breast cancer models, murine EMT-6 and human MCF-7 and MDA-MB-231 tumor tissue homogenates (*left*) and cell lysates (*right*)

Protein expression of LOX was studied in EMT-6, MCF-7 and MDA-MB-231 cells and tissue homogenates. The results depicted in Fig. 3 confirm expression of LOX in all cell lines studied.

LOX expression was verified by Western blot analysis on tumor homogenates and cell lines. The high expression level of LOX in all cell lines and tumor tissue homogenates is an important finding for subsequent molecular targeting experiments of LOX using [^18^F]FB-GGGDPKGGGGG-NH_2_ and FITC-Ava-GGGDPKGGGGG-NH_2_.

### LOX protein expression under normoxic and hypoxic conditions using confocal microscopy and flow cytometry

**Fig. 4:**
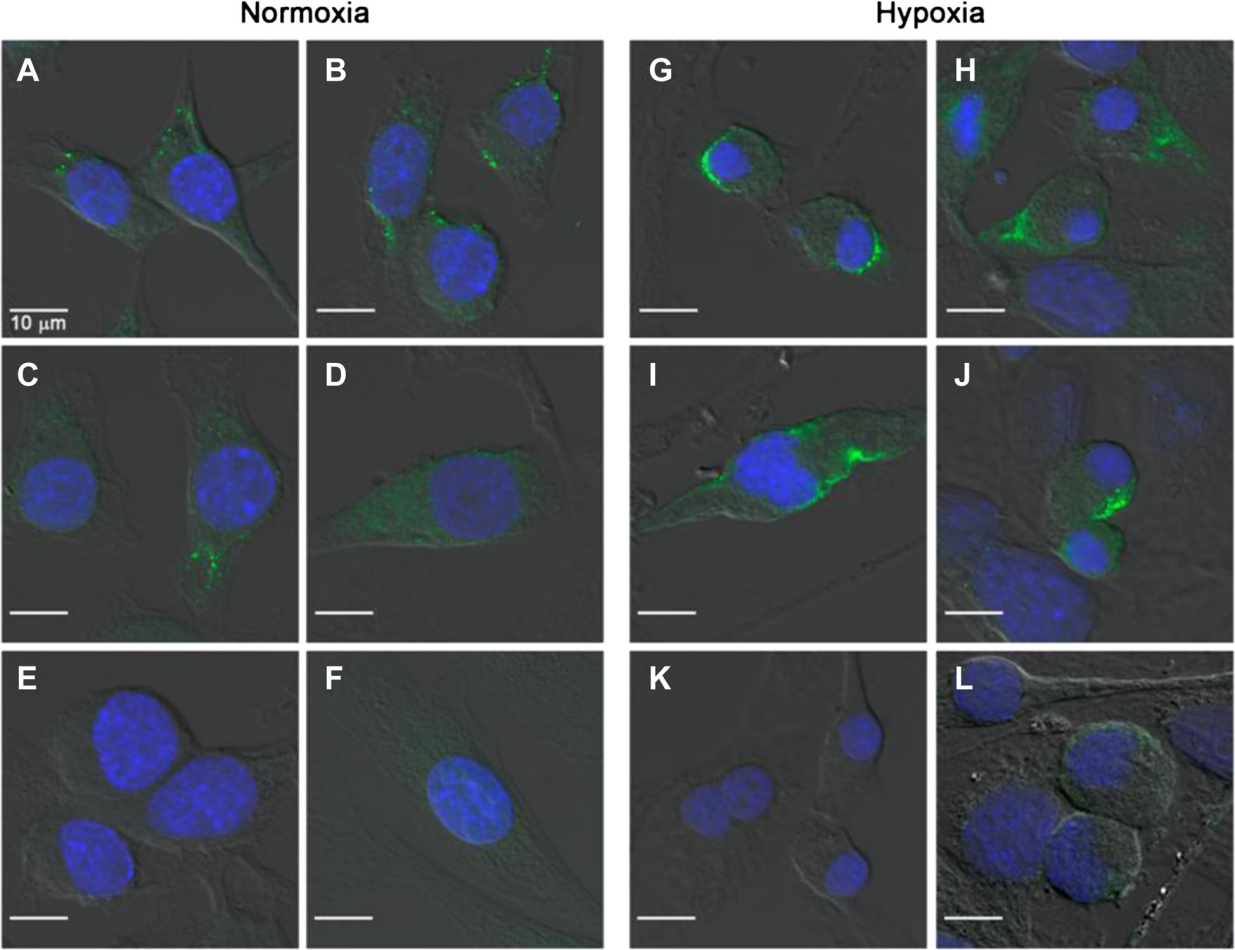
Confocal microscopy images of EMT-6 cells cultured under (**a**–**d**) normoxic and (**g**–**j**) hypoxic conditions using primary anti-lysyl oxidase (anti-LOX) monoclonal antibody (mAb) and a secondary Alexa Fluor 488 mAb. Cell nuclei were stained with 4′,6-diamidino-2-phenylindole. Control experiments in which (**e**, **k**) only the primary LOX mAb and (**f**, **l**) only secondary Alexa Fluor 488 mAb were used are shown

To be able to investigate the potential binding and interaction of the peptide sequence GGGDPKGGGGG with the LOX enzyme, murine EMT-6 breast cancer cells were analyzed for their binding of primary anti-LOX antibody by confocal microscopy. Figure 4 summarizes the results of these experiments under normoxic and hypoxic conditions.

**Fig. 5:**
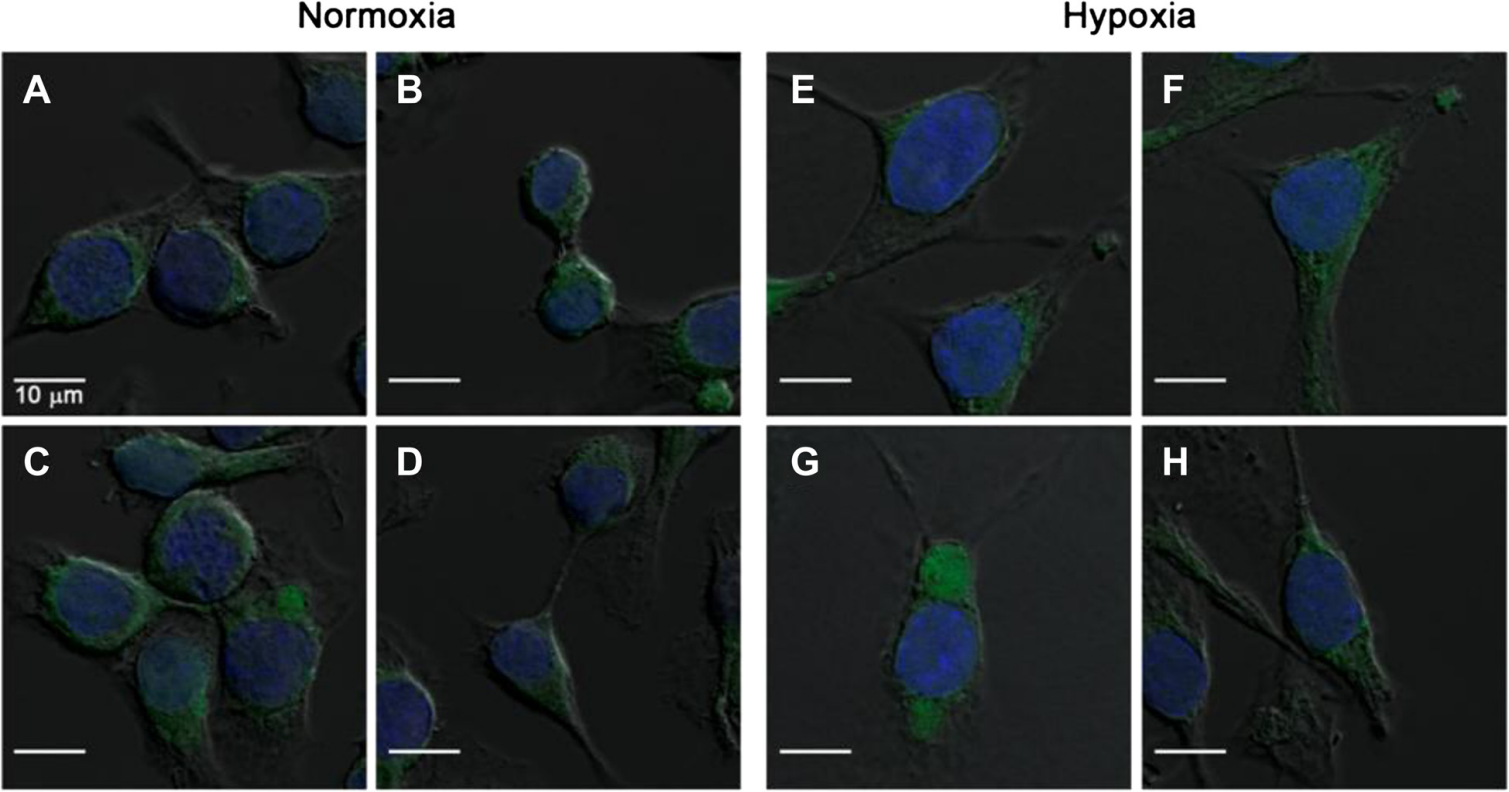
Confocal microscopic images of EMT-6 cells cultured under (**a**–**c**) normoxic and (**e**–**g**) hypoxic conditions using fluorescein isothiocyanate (FITC)-Ava-GGGDPKGGGGG-NH_2_. Cell nuclei were stained with 4′,6-diamidino-2-phenylindole. (**d**, **h**) The results of control experiments without FITC-Ava-GGGDPKGGGGG-NH_2_

Hypoxic conditions were chosen because it has been shown that higher expression of LOX may correlate with a higher uptake of ^18^F-labeled fluoroazomycin arabinoside ([^18^F]FAZA), a PET radiotracer for imaging tumor hypoxia [37]. Under normoxic conditions, the LOX antibody readily bound to some areas within the EMT-6 cell. Under hypoxia, much larger areas with fluorescent signaling resulting from binding of the LOX-specific antibody were observed in parts of the cell membrane and at opposite poles in dividing EMT-6 cells. The observations indicated an increase in protein expression under hypoxia. In a second experiment in which we used FITC-Ava-GGGDPKGGGGG-NH_2_ incubated with the EMT-6 cells, the peptide was found to bind with the inner compartments of cells, which may include binding to LOX as well as non-specific interactions with other off-target proteins (Fig. 5).

**Fig. 6:**
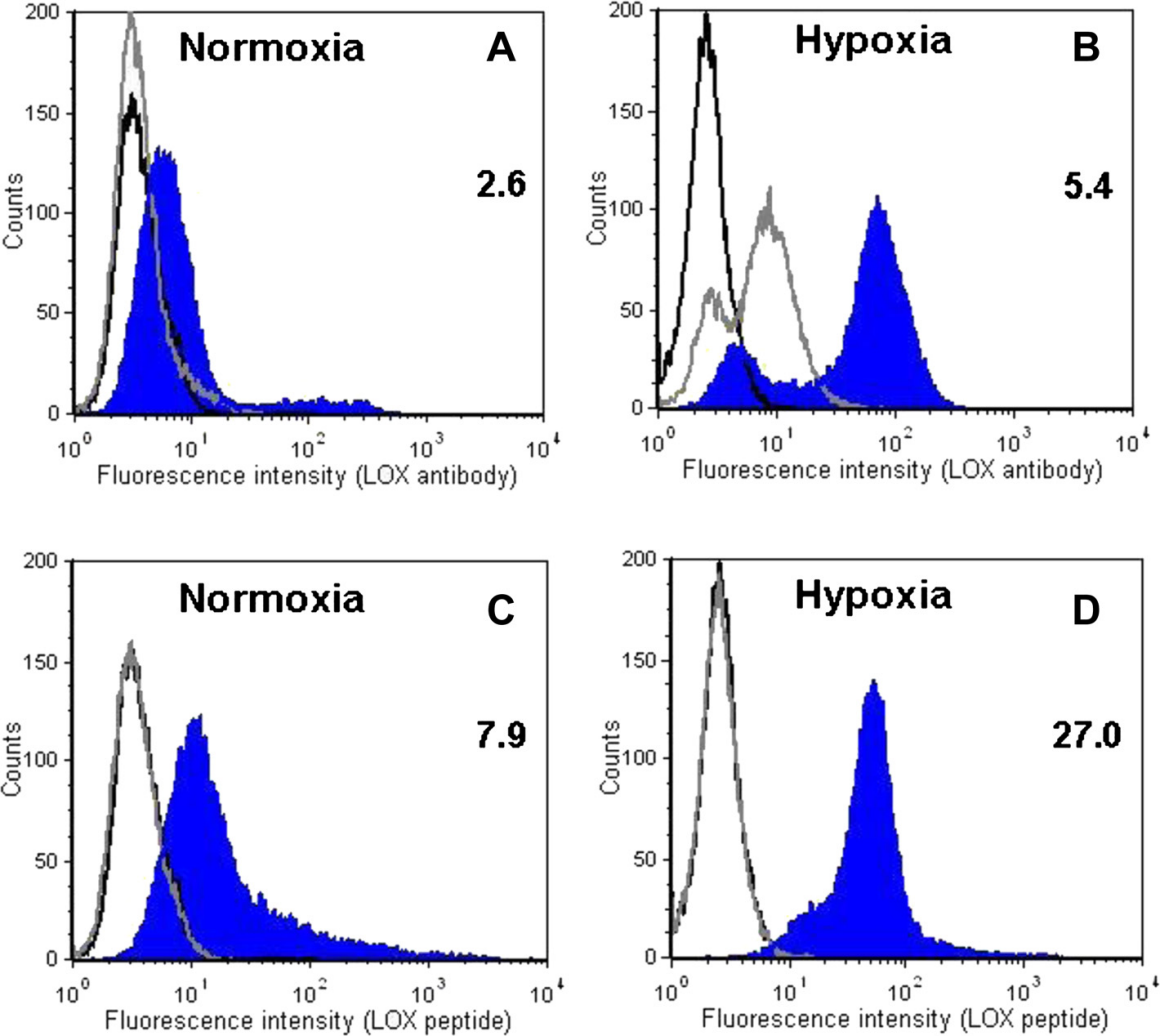
Flow cytometry of lysyl oxidase (LOX) in EMT-6 cells cultured under (**a**, **c**) normoxic conditions versus (**b**, **d**) hypoxic conditions. Cells were treated with primary anti-LOX antibody, followed by (**a**, **b**) staining with Alexa Fluor 488–labeled secondary antibody or (**c**, **d**) treatment with fluorescein isothiocyanate (FITC)-Ava-GGGDPKGGGGG-NH_2_. The *x*- and *y*-axes of each graph show the fluorescence intensity and the number of cells, respectively. Representative results of three experiments are shown. The *black open* histogram depicts unstained cells. Values in each panel are the ratio of tumor volume in control (C) versus treated (T) mice calculated based on the mean fluorescence intensity of *blue closed* (T: LOX expression detected with anti-LOX antibody and FITC-Ava-GGGDPKGGGGG-NH_2_, respectively) and *gray open* (C: only Alexa Fluor 488–labeled secondary antibody control and GGGDPKGGGGG-NH_2_ without FITC-labeled control, respectively) histograms (n = 3)

Fluorescence staining with the FITC-labeled peptide appeared to be more perinuclear than membrane-localized, as was true with the specific anti-LOX antibody (see Fig. 4, *right*). For a more quantitative approach flow cytometry analysis was performed under normoxic and hypoxic conditions (Fig. 6).

Under normoxic conditions, both the specific anti-LOX antibody plus the Alexa Fluor 488–labeled secondary antibody and FITC-Ava-GGGDPKGGGGG-NH_2_ induced a fluorescent signal, indicating binding to EMT-6 cells, whereas under hypoxic conditions, fluorescence intensities, and therefore cellular binding, increased considerably. In case of the LOX-specific antibody, the mean fluorescence increased about twofold, from 2.6 to 5.4. With FITC-Ava-GGGDPKGGGGG-NH_2_, a threefold rise in mean fluorescence intensity, from 7.9 to 27.0, was observed.

### Radiosynthesis and in vitro cell uptake of radiolabeled peptide

**Fig. 7:**
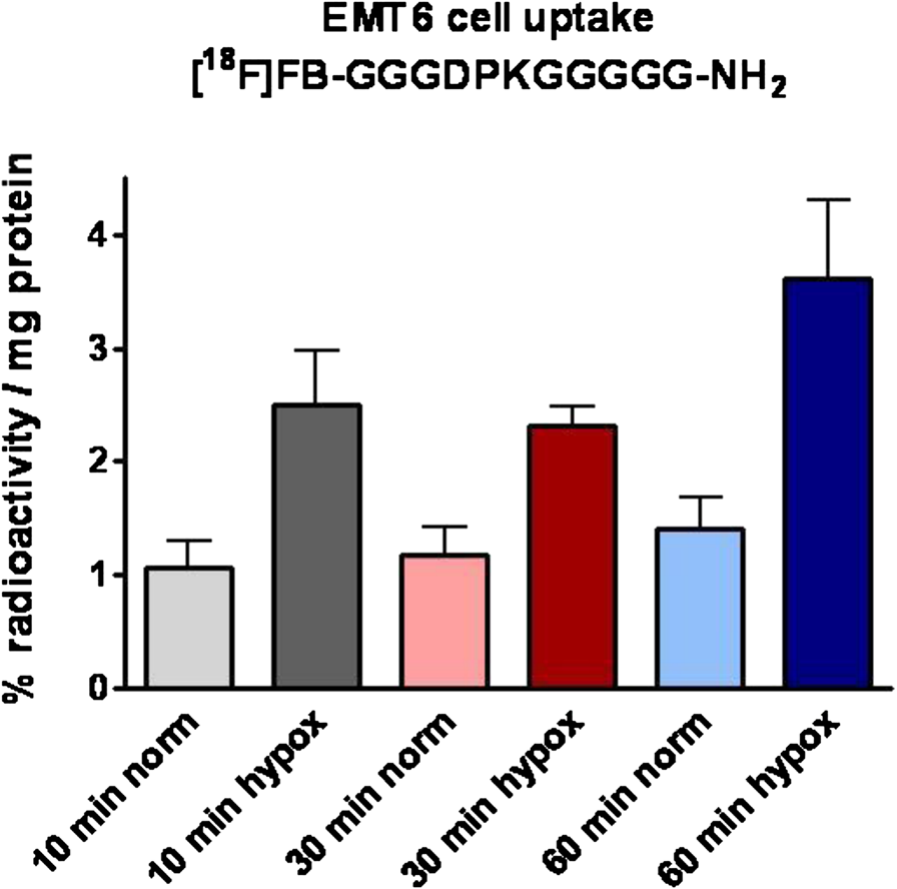
Uptake of [^18^F]fluorobenzoate ([^18^F]FB)-GGGDPKGGGGG-NH_2_ into EMT-6 cells under normoxic versus hypoxic conditions after 10-, 30- and 60-min incubation times. Data are shown as the percentage radioactivity normalized to milligrams of protein and as mean ± standard error of the mean from three different experiments

Radiosynthesis of [^18^F]FB-GGGDPKGGGGG-NH_2_ was achieved in good radiochemical yields (18–50 %, decay-corrected) and with high radiochemical purity (>98 %). We used solid-phase extraction, cartridge-purified [^18^F]SFB according to our long-term standard procedure [38], which we have optimized for labeling peptides [39]. To see if the radiolabeled peptide would bind or show any cellular uptake compared with the in vitro results using the FITC-labeled peptide, cellular uptake studies were carried out with EMT-6 cells under normoxic and hypoxic conditions (Fig. 7).

Under normal cell culture conditions, radioactivity in the cells was found at only 1.07±0.24 (*n* = 6/3) after 10-min incubation and 1.41±0.27 % radioactivity / mg protein (*n* = 4/2) after 60-min incubation. However, during hypoxia, the amount of [^18^F]FB-GGGDPKGGGGG-NH_2_ detected in the cells was increased significantly, reaching 2.50±0.47 (*n* = 6/3; *p* < 0.05) after 10-min incubation and 3.62±0.69 % radioactivity / mg protein (*n* = 4/2; *p* < 0.05) after 60-min incubation. This in vitro result with the radiolabeled peptide confirmed our observations with the FITC-labeled peptide in that, in both cases, quantitatively more of the peptide was found in the EMT-6 cells under hypoxic conditions.

### Radiolabeled peptide for imaging LOX in vivo metabolic stability

**Fig. 8:**
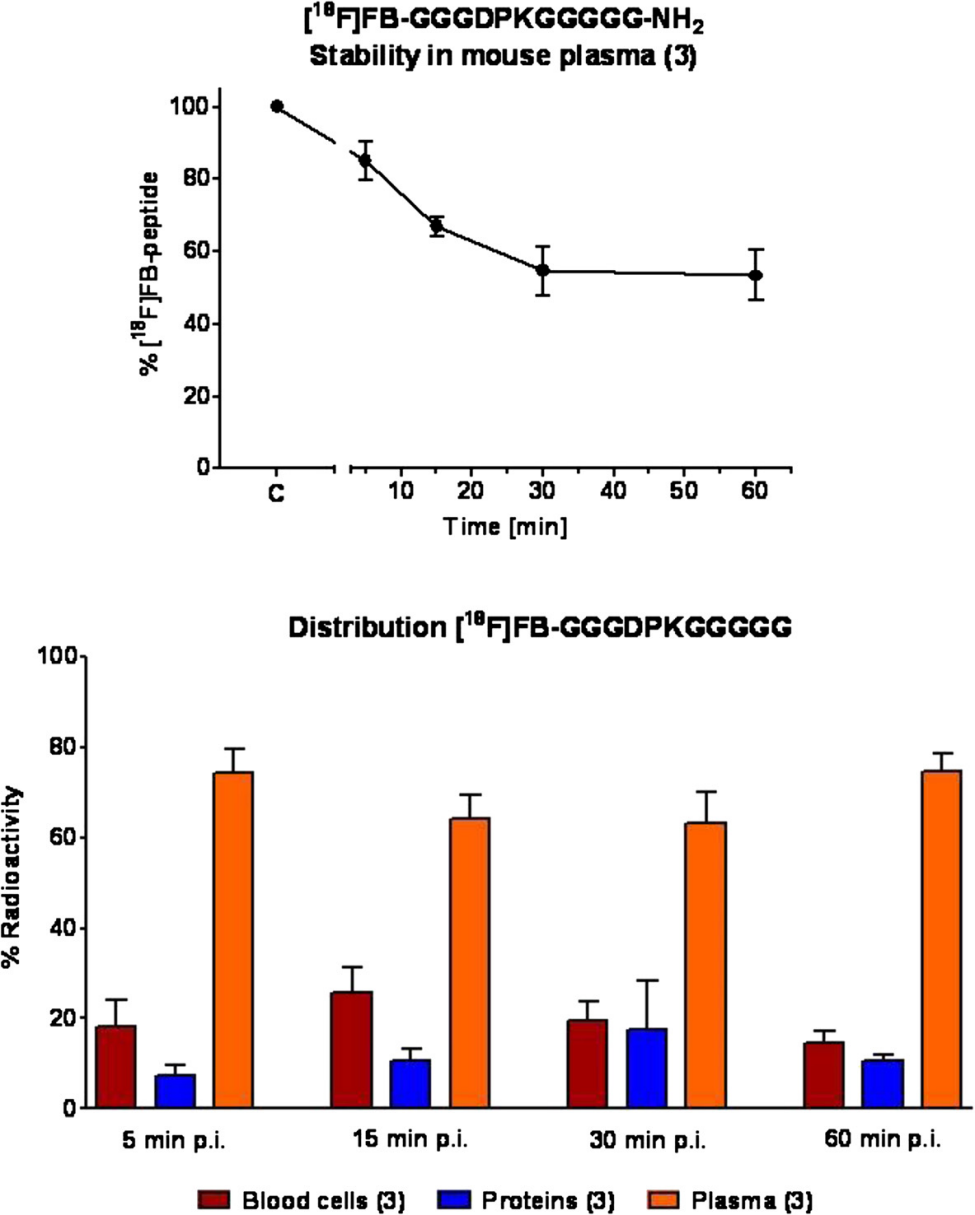
Stability of [^18^F]fluorobenzoate ([^18^F]FB)-GGGDPKGGGGG-NH_2_ in mouse plasma over the course of 60 min postinjection (p.i.) (*top*). Distribution of radioactivity into the three blood compartments (blood cells, plasma proteins and plasma) at 5, 15, 30 and 60 min after injection of the radiolabeled peptide (*bottom*). Data are shown as mean ± standard error of the mean from three experiments

As a next step, the in vivo stability of the radiolabeled peptide was tested in normal BALB/c mice. After 5 min, 85±5 % (*n* = 3) of the injected radiolabeled peptide was found to be intact. This amount decreased over the course of 30 min postinjection to 55±7 % (*n* = 3), but it remained almost unchanged over the next 30 min. After 60 min postinjection, 53±7 % (*n* = 3) of intact [^18^F]FB-GGGDPKGGGGG-NH_2_ could be detected in mouse plasma (Fig. 8, *top*). Whereas quality control of [^18^F]FB-GGGDPKGGGGG-NH_2_ resulted in a retention time (*t*_0_) of 12.2 min for the injected radiotracer under the chosen HPLC conditions, two different, more lipophilic radiometabolites were detected (*t*_R_ = 13.2 and 14.3 min). Overall, most of the injected [^18^F]FB-GGGDPKGGGGG-NH_2_ (>60 %) was found in the plasma compartment, and a minority of the radiotracer was bound to blood cells or plasma proteins (Fig. 8, *bottom*).

### Dynamic PET imaging in tumor-bearing mice

**Fig. 9:**
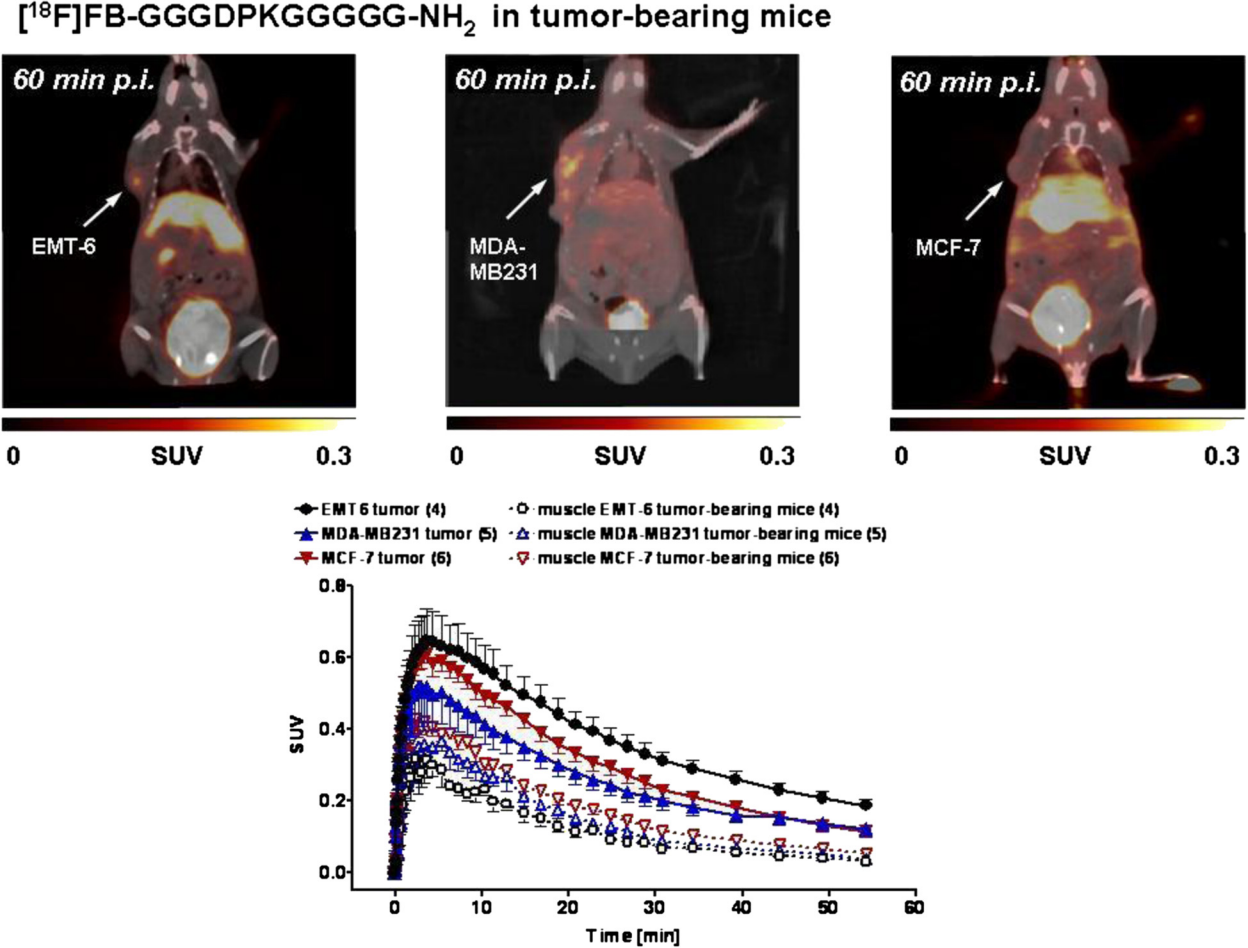
*Top:* Representative positron emission tomography/computed tomography of EMT-6, MDA-MB-231 and MCF-7 tumor-bearing mice 60 min after injection of [^18^F]fluorobenzoate ([^18^F]FB)-GGGDPKGGGG-NH_2_. Images are shown as maximum intensity projections. *Bottom:* Time–activity curves for the radioactivity detected in EMT-6, MDA-MB-231 and MCF-7 tumors in comparison with the uptake levels in muscle tissue after injection of [^18^F]FB-GGGDPKGGGGG-NH_2_. Data are presented as mean ± standard error of the mean. *p.i.* postinjection, *SUV* standardized uptake value

[^18^F]FB-GGGDPKGGGGG-NH_2_ was injected into three mouse tumor models: the murine model (EMT-6) and the two human xenograft models (MCF-7 and MDA-MB-231). In all three tumor types, initial uptake of radioactivity was observed, leading to maximum SUV values after 3–5 min postinjection. SUV_5min_, values were 0.63±0.08 (*n* = 4) in EMT-6, 0.59±0.03 (*n* = 6) in MCF-7 and 0.50±0.09 (*n* = 5) in MDA-MB-231. However, following the rapid initial uptake, a continuous washout of the radioactivity was observed over the following 60 min postinjection. SUV_60min_ values were 0.19±0.02 (*n* = 4) in EMT-6, 0.12±0.02 (*n* = 6) in MCF-7 and 0.12±0.02 (*n* = 5) in MDA-MB-231 (Fig. 9).

The determined radioactivity in muscle tissue was always lower than the tumor values: SUV_60min_ values were 0.03±0.004 (*n* = 4; EMT-6), 0.05±0.008 (*n* = 6; MCF-7) and 0.04±0.004 (*n* = 5; MDA-MB-231). Whereas the tumor-to-muscle ratio decreased from 2.7 after 5 min to 1 after 60 min, the tumor-to-blood ratio increased from 0.3 to 2.3 based on the blood clearance of the injected radioactivity. Overall, comparable maximum radioactivity uptake levels were found in all three breast cancer tumor models analyzed: the murine EMT-6 and the human MCF-7 and MDA-MB-231 cell lines (Fig. 9).

### Effects of BAPN, an irreversible blocker of LOX

**Fig. 10:**
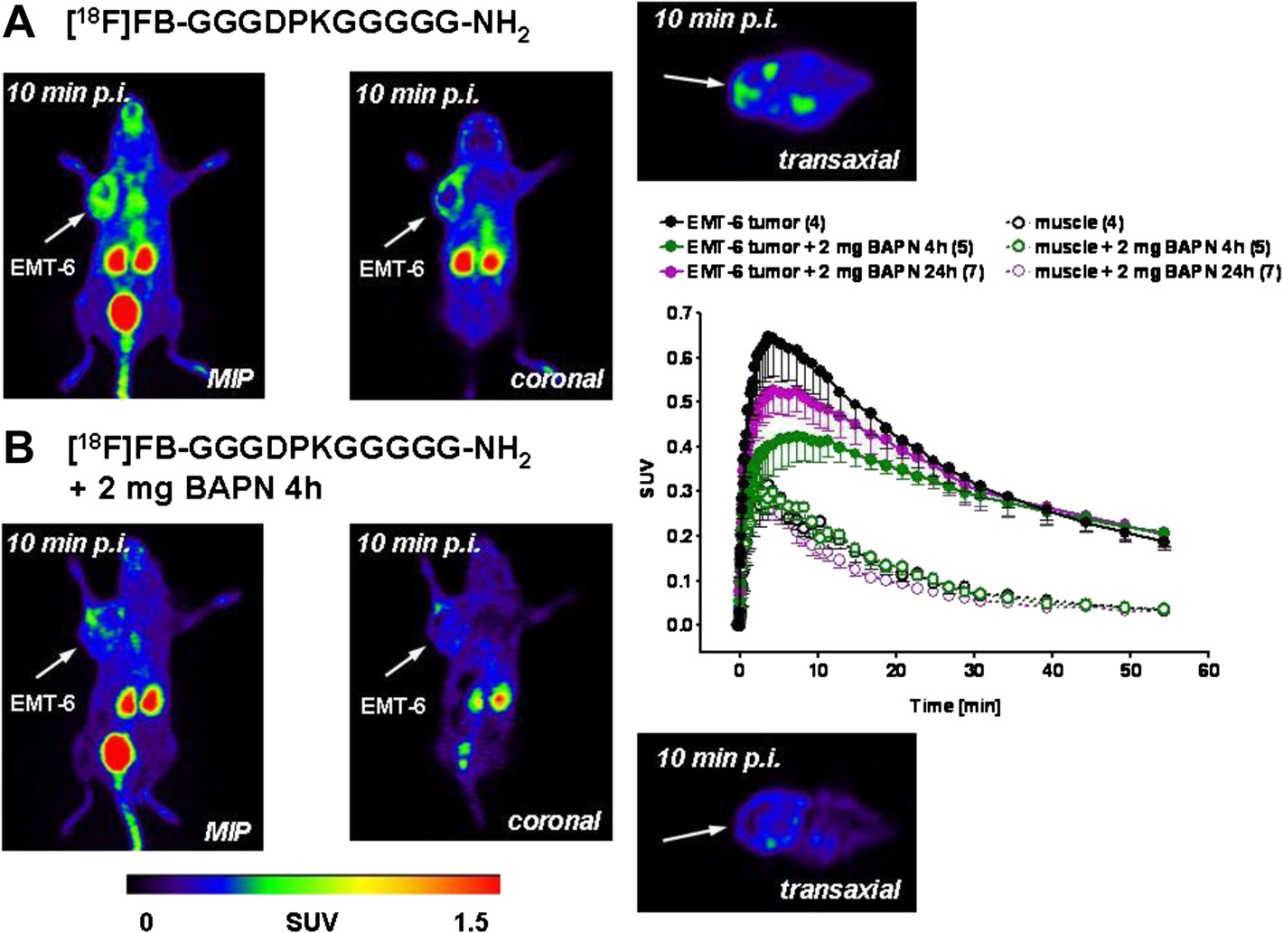
Representative positron emission tomographic images after injection (p.i.) of [^18^F]fluorobenzoate ([^18^F]FB)-GGGDPKGGGGG-NH_2_ in the absence and presence of β-aminopropionitrile (BAPN) in EMT-6 tumor-bearing mice at 10 min p.i. Images are shown as maximum intensity projections (MIPs) and coronal and transaxial slices from the tumor region. *Right*: Time–activity curves for the radioactivity uptake into EMT-6 tumors in comparison with muscle tissue in the absence and presence of BAPN. Data are presented as mean ± standard error of the mean. *SUV* standardized uptake value

The EMT-6 tumor model was chosen to study the tumor uptake of the radiolabeled peptide sequence in the presence and absence of BAPN. This compound is an irreversible blocker of LOX [20]. To date, it is the most potent known inhibitor of LOX, and it is used as the reference compound for the inhibition of LOX activity [22, 25]. Figure 10 presents PET images obtained at 10 min after injection of [^18^F]FB-GGGDPKGGGGG-NH_2_, where BAPN was injected 24 h before the radiotracer and the creation of the summarized TACs for the radioactivity uptake into tumor and muscle tissue in the presence and absence of BAPN.

Intraperitoneal injection of 100 mg/kg BAPN (corresponding to about 2 mg per mouse) in 100 μl of PBS 4 h before intravenous injection of [^18^F]FB-GGGDPKGGGGG-NH_2_ reduced its maximum uptake levels into the tumor from SUV_5min_ 0.63±0.08 in the absence of BAPN to 0.42±0.06 (*n* = 4) in the presence of BAPN (*n* = 5) (*p* = 0.076), amounting to a reduction of approximately 33 %.

After 24-h preinjection of BAPN, the inhibitory effects tended to be slightly lower: SUV_5min_ 0.52±0.05 (*n* = 7; *p* > 0.05). However, at 60 min postinjection, the uptake levels reached similar low values (SUV_60min_ = 0.19–0.21), indicating that there was no effect of BAPN. Observed radioactivity in muscle tissue was always below tumor uptake levels with a faster washout and was unaltered in the presence of BAPN (Fig. 10). The latter observation supports the suggestion that the observed effects of BAPN on radioactivity tumor uptake after injection of [^18^F]FB-GGGDPKGGGGG-NH_2_ were indeed tumor-specific.

## Discussion

The following are the main findings of this study:

1. The LOX protein is expressed in all three murine and human breast cancer cell lines investigated, as well as in the tumors generated from these cells. Furthermore, confocal microscopy of EMT-6 cells with a LOX-specific antibody provided evidence for the membrane association of the enzyme.
2. Hypoxia leads to an increase in LOX protein expression at the cellular level, as shown in EMT-6 cells with use of the specific LOX antibody.
3. FITC-Ava-GGGDPKGGGGG-NH_2_ binds to several cell compartments, which may include those containing LOX.
4. After injection of radiolabeled [^18^F]FB-GGGDPKGGGGG-NH_2_, tumor uptake in all three models of breast cancer was detected.
5. Tumor uptake tended to be reduced during the initial phase after radioactivity injection when the irreversible LOX inhibitor BAPN was present.

The patient mRNA data derived from this study confirm the correlation between HIF-1α and LOX expression. High LOX expression is related to a poor breast cancer patient outcome. TNBCs display higher LOX expression, and reports in the literature suggest that LOX can be used as a prognostic marker for breast cancer [12]. In recent years, research focus has shifted toward the LOXL2 isoform owing to its elevated expression in patients with breast cancer [40]. Interestingly, LOXL4 displays a very different mRNA expression profile, which may be associated with a different, yet unknown biological role in breast cancer. To date, knowledge about the precise molecular function of LOXL4 in tumors is very limited, even though links to tumor progression are established [41–43]. It was demonstrated that splice variants may be responsible for different effects on tumor progression and tumor suppression. Moreover, LOX itself was discussed to possess a role in both effects in colon cancer [41]. This confirms the complexity of the regulation of LOX and the other enzyme family members. The high levels of LOX in breast cancer and its important role during metastasis make it an important target for non-invasive molecular imaging in vivo.

The LOX active site small-molecule antagonist BAPN has been investigated for a long time as a potential drug for cancer therapy [15–17, 22, 23]. Among various amine oxidases, LOX is selectively inhibited by this substance [44, 45]. However, systemic administration of BAPN leads to physiological side effects, such as blood pressure reduction, which seem to be LOX-mediated [24]. To allow a specific delivery of BAPN to hypoxic regions in tumors, a prodrug approach based on reductive activation has been pursued [20]. The development of a LOX-targeting imaging probe requires a LOX-specific binding molecule. To date, except for BAPN, no small-molecule inhibitors targeting the LOX enzyme family with more favorable pharmacological properties have been developed [20].

An alternative approach for specific targeting of LOX involves the use of oligopeptides [31]. Nagan and Kagan have investigated collagen-like structures to explore peptide sequences derived from the lysine-containing N- and C-terminal telopeptides of collagen concerning their enzymatic conversion by LOX. They found that the peptide sequence GGGDPKGGGGG displayed the lowest Michaelis constant (*K*_m_) among several collagen N-telopeptide derivatives, resulting in the highest *k*_cat_/*K*_m_, as determined through the peptidyl lysine oxidation. It was concluded that the aspartic acid located two positions N-terminal to the lysine residue is critical for binding to LOX and the subsequent catalytic processing. For the present study, this specific peptide sequence was selected for radiolabeling with the short-lived positron emitter ^18^F.

Peptides can be commonly labeled with prosthetic groups containing ^18^F according to standard procedures [32, 35, 46]. Peptide [^18^F]FB-GGGDPKGGGGG-NH_2_ was very stable over the course of 60 min postinjection in vivo. High radioactivity accumulation was found in all three selected breast cancer models during the first 10 min of a dynamic PET experiment. However, on the one hand, a washout was observed over time, which is indicative of no trapping of radioactivity in the tumor tissue. On the other hand, over time, the radioactivity detected in the tumor tissue was always higher than in muscle background tissue.

In our own studies, we previously demonstrated that EMT-6 tumors display a defined level of hypoxia-driven uptake of [^18^F]FAZA [47]. Hypoxic breast cancer cells also express higher levels of LOX, as demonstrated in a previous study with human MDA-MB-231 cells [11]. This finding was confirmed by confocal microscopy and flow cytometry experiments in EMT-6 cells using a LOX-specific antibody that indicated elevated LOX levels in the analyzed breast cancer cells under hypoxic conditions. Uptake of [^18^F]FB-GGGDPKGGGGG-NH_2_ into EMT-6 cells was also elevated under hypoxic conditions, confirming that uptake and/or binding of this radiolabeled peptide is somewhat dependent on hypoxia and that specific binding sites are present on or within these breast cancer cells. The presence of the specific and irreversible LOX blocker BAPN [23] led to a reduction of [^18^F]FB-GGGDPKGGGGG-NH_2_ uptake into EMT-6 tumors within the first 30 min postinjection. However, at 60 min postinjection, no differences were detectable between control and blocked radioactivity levels in the tumors. This suggests that a specific interaction of [^18^F]FB-GGGDPKGGGGG-NH_2_ with LOX in the tumor tissue may occur, but only at the beginning of the dynamic observation. Specific interaction is further supported by the fact that, in muscle reference tissue, no effect upon preadministration of BAPN was seen. In addition to the findings of Nagan and Kagan that GGGDPKGGGGG binds to LOX in vitro [31], the present experiments suggest that FITC-GGGDPKGGGGG may also possess some non-specific binding in EMT-6 cells, in addition to binding to LOX. Therefore, tumor uptake and blocking effects in vivo in the presence of BAPN indicate both a specific interaction with LOX and non-specific processes.

## Conclusions

The present data warrant further investigations devoted to the development of LOX-specific binding compounds for molecular imaging of LOX in vivo. The role of specific targeting of LOX using molecular imaging techniques may be used for breast cancer staging and monitoring responses to therapy.

## Abbreviations

Ava: 5-Aminovaleric acid
BAPN: β-Aminopropionitrile
BCA: Bicinchoninic acid
CT: Computed tomography
DAPI: 4′,6-Diamidino-2-phenylindole
DMF: Dimethylformamide
ECM: Extracellular matrix
EDTA: Ethylenediaminetetraacetic acid
ER: Estrogen receptor
ESI: Electron spray ionization
^18^F: Fluorine-18
FACS: Fluorescence-activated cell sorting
[^18^F]FAZA: ^18^F-labeled fluoroazomycin arabinoside
[^18^F]FB: [^18^F]fluorobenzoate
[^18^F]FDG: 2-deoxy-2-[^18^F]fluoro-d-glucose
[^18^F]FES: 16α-[^18^F]fluoro-17β-estradiol
FITC: Fluorescein isothiocyanate
[^18^F]FLT: 3-Deoxy-3-[^18^F]fluorothymidine
[^18^F]SFB: *N*-succinimidyl-4-[^18^F]fluorobenzoate
HIF-1α: Hypoxia-inducible factor 1α
HPLC: High-performance liquid chromatography
IgG: Immunoglobulin G
LOX: Lysyl oxidase
mAb: Monoclonal antibody
MBHA: 4-Methylbenzhydrylamine
MIP: Maximum intensity projection
MS: Mass spectrometry
PBS: Phosphate-buffered saline
PET: Positron emission tomography
ROI: Region of interest
SEM: Standard error of the mean
SPPS: Solid-phase peptide synthesis
SUV: Standardized uptake value
TAC: Time–activity curve
TBST: Tris-buffered saline containing 0.05 % (v/v) Tween 20
CA: Trichloroacetic acid
TFA: Trifluoroacetic acid
TIPS: Triisopropylsilane
TMA: Tissue microarray
TNBC: Triple-negative breast cancer
